# Roles of plastid-located phosphate transporters in carotenoid accumulation

**DOI:** 10.3389/fpls.2022.1059536

**Published:** 2022-12-15

**Authors:** Dong-Li Hao, Jin-Yan Zhou, Ya-Nan Huang, Hao-Ran Wang, Xiao-Hui Li, Hai-Lin Guo, Jian-Xiu Liu

**Affiliations:** ^1^ The National Forestry and Grassland Administration Engineering Research Center for Germplasm Innovation and Utilization of Warm-Season Turfgrasses, Jiangsu Key Laboratory for the Research and Utilization of Plant Resources, Institute of Botany, Jiangsu Province and Chinese Academy of Sciences (Nanjing Botanical Garden Mem. Sun Yat-Sen), Nanjing, China; ^2^ Department of Agronomy and Horticulture, Jiangsu Vocational College of Agriculture and Forest, Jurong, China; ^3^ Co-Innovation Center for Sustainable Forestry in Southern China, College of Biology and the Environment, Nanjing Forestry University, Nanjing, China

**Keywords:** plastid, chromoplast, chloroplast, phosphate transporter, carotenoid accumulation

## Abstract

Enhanced carotenoid accumulation in plants is crucial for the nutritional and health demands of the human body since these beneficial substances are acquired through dietary intake. Plastids are the major organelles to accumulate carotenoids in plants and it is reported that manipulation of a single plastid phosphate transporter gene enhances carotenoid accumulation. Amongst all phosphate transport proteins including phosphate transporters (PHTs), plastidial phosphate translocators (pPTs), PHOSPHATE1 (PHO1), vacuolar phosphate efflux transporter (VPE), and Sulfate transporter [SULTR]-like phosphorus distribution transporter (SPDT) in plants, plastidic PHTs (PHT2 & PHT4) are found as the only clade that is plastid located, and manipulation of which affects carotenoid accumulation. Manipulation of a single chromoplast PHT (PHT4;2) enhances carotenoid accumulation, whereas manipulation of a single chloroplast PHT has no impact on carotenoid accumulation. The underlying mechanism is mainly attributed to their different effects on plastid orthophosphate (Pi) concentration. PHT4;2 is the only chromoplast Pi efflux transporter, and manipulating this single chromoplast PHT significantly regulates chromoplast Pi concentration. This variation subsequently modulates the carotenoid accumulation by affecting the supply of glyceraldehyde 3-phosphate, a substrate for carotenoid biosynthesis, by modulating the transcript abundances of carotenoid biosynthesis limited enzyme genes, and by regulating chromoplast biogenesis (facilitating carotenoid storage). However, at least five orthophosphate influx PHTs are identified in the chloroplast, and manipulating one of the five does not substantially modulate the chloroplast Pi concentration in a long term due to their functional redundancy. This stable chloroplast Pi concentration upon one chloroplast PHT absence, therefore, is unable to modulate Pi-involved carotenoid accumulation processes and finally does affect carotenoid accumulation in photosynthetic tissues. Despite these advances, several cases including the precise location of plastid PHTs, the phosphate transport direction mediated by these plastid PHTs, the plastid PHTs participating in carotenoid accumulation signal pathway, the potential roles of these plastid PHTs in leaf carotenoid accumulation, and the roles of these plastid PHTs in other secondary metabolites are waiting for further research. The clarification of the above-mentioned cases is beneficial for breeding high-carotenoid accumulation plants (either in photosynthetic or non-photosynthetic edible parts of plants) through the gene engineering of these transporters.

## 1 Introduction

Carotenoids are a kind of colorful C40 lipophilic isoprenoid pigments naturally found in plants (Cazzonelli and Pogson, 2010; Ohmiya et al., 2019). They serve as critical components for photosynthesis and play key roles in the photoprotection of photosynthetic machinery (Green and Durnford, 1996; Niyogi, 2000; Pogson et al., 2005; Li et al., 2009; Domonkos et al., 2013; Niyogi and Truong, 2013; Hashimoto et al., 2016; Osorio, 2019; Sun and Li, 2020). They provide precursors for the biosynthesis of phytohormones such as abscisic acids (ABA) and strigolactones (SLs), consequently regulating plant growth and development (Nambara and Marion-Poll, 2005; DellaPenna and Pogson, 2006; Howitt and Pogson, 2006; Gomez-Roldan et al., 2008; Umehara et al., 2008; Cazzonelli and Pogson, 2010; Al-Babili and Bouwmeester, 2015; Jia et al., 2018). Carotenoid derivatives also act as signaling molecules to modulate plant development and responses to environmental stimuli (Havaux, 2014; Tian, 2015; Hou et al., 2016; Sun et al., 2018; Dickinson et al., 2019; Feder et al., 2019; Sun and Li, 2020; Sierra et al., 2022).

Apart from the above-mentioned roles in plants, carotenoids play critical roles in human nutrition and health as essential components of human diets. They provide provitamin A and serve as antioxidants to reduce the incidence of some chronic diseases, such as cardiovascular diseases, cancers, and age-related eye diseases (Fraser and Bramley, 2004; Rao and Rao, 2007; Hannoufa and Hossain, 2012; Fiedor and Burda, 2014). The vivid yellow, orange, and red colors, which are endowed with high levels of carotenoid accumulation, are not only an important quality trait for fruits and vegetables but also a critical agronomic trait for fruits and flowers in many horticultural crops (Egea et al., 2010; Li and Yuan, 2013; Yuan et al., 2015; Schweiggert and Carle, 2017; Ohmiya et al., 2019; Hermanns et al., 2020). In addition, some carotenoids are used as supplements in livestock and fish feed formulations, and also as natural colorants in the food and cosmetic industries (Umeno et al., 2005).

The pivotal role of carotenoids in plants combined with high market demands has triggered extensive research into enhancing carotenoid accumulation in plants (Farré et al., 2011; Li and Yuan, 2013; Nisar et al., 2015). Plastids are the organelles/sites for carotenoid biosynthesis and storage in plant cells (Sun et al., 2018; Hermanns et al., 2020). In detail, chromoplasts of roots, fruits, and flower petals (non-photosynthetic tissues) and chloroplasts of green tissues (photosynthetic tissues) are the major plastids to accumulate carotenoids in plants (Cazzonelli and Pogson, 2010; Ruiz-Sola and Rodrı´guez-Concepcio´n, 2012; Li and Yuan, 2013; Nisar et al., 2015; Li L. et al., 2016; Sun et al., 2018; Hermanns et al., 2020). Increasing carotenoid accumulation by manipulating key genes that are directly involved in carotenoid biosynthesis (such as phytoene synthase, and phytoene desaturase), has been extensively studied and many successful advances have been made (Lu and Li, 2008; Cazzonelli and Pogson, 2010; Hannoufa and Hossain, 2012; Yuan et al., 2015; Feder et al., 2019; Ohmiya et al., 2019; Osorio, 2019; Hermanns et al., 2020; Luan et al., 2020; Sathasivam et al., 2020). The above-mentioned strategy (using a single gene) powerfully enhances one or two specific carotenoids in plants by regulating a specific process involved in carotenoid accumulation, whereas its contribution to the simultaneous enhancement of several kinds of carotenoids is largely restricted (Römer et al., 2000; Ralley et al., 2004; Enfissi et al., 2005; Fraser et al., 2007; Simkin et al., 2007; Suzuki et al., 2007; Wurbs et al., 2007; Jayaraj et al., 2008; Apel and Bock, 2009; Ha et al., 2010; D'Ambrosio et al., 2011; Schmidt et al., 2015; Paul et al., 2017; McQuinn et al., 2018; Yao et al., 2018; Hermanns et al., 2020; Zheng et al., 2020). Recently, overexpression of a chromoplast-located phosphate transporter (PHT), rather than a protein that is an element for carotenoid biosynthesis, enhances carotenoid accumulation in plants through co-enhancement of at least four types of carotenoids (Zhang et al., 2017; Lu et al., 2018), providing a new strategy for comprehensive enhancement of carotenoids. Surprisingly, the manipulation of a single PHT from another type of plastid, chloroplast, does not affect carotenoid accumulation in photosynthetic tissues (Karlsson et al., 2015; Miyaji et al., 2015).

**Figure 1:**
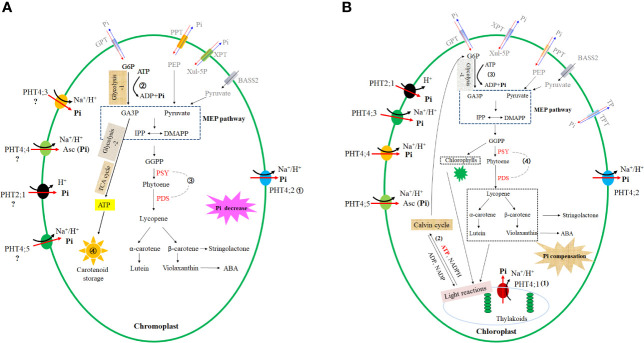
Roles of plastid-located PHTs in carotenoid accumulation. **(A)** Underlying mechanisms for the observation that overexpression of single chromoplast PHT enhances carotenoid accumulation in non-photosynthetic tissues. ① Overexpression of a major chromoplast phosphate exporter (PHT4;2) results in accelerated phosphate efflux from the chromoplast. ② The enhanced phosphate efflux from chromoplast facilitates ATP hydrolysis, promoting the energy supply required for the process of substrate synthesis (GA3P) required for carotenoid biosynthesis. ③ The lower phosphate concentration in chromoplast upregulates the transcriptional abundances of carotenoid biosynthesis key genes (*PSY, PDS*). ④ Accelerated phosphate efflux facilitates chromoplast development, favoring carotenoid storage. The chromoplast location of PHT2;1, PHT4;3, PHT4;4, PHT4;5 is obtained by speculation, which case needs further experimental pieces of evidence. **(B)** Underlying mechanisms for the observation that manipulation of single chloroplast PHT does not affect the carotenoid accumulation in photosynthetic tissues. (1) The functional redundancy amongst at least five chloroplast PHTs causes the result that manipulation of a single chloroplast PHT does not substantially affect the phosphate concentration in the chloroplast. This case consequently leads to the inability to affect processes (2) to (4). (2) As the basis for the photosynthesis of plants, photosynthetic pigments (chlorophylls and carotenoids) are crucial for the generation of ATP and NADPH at the light reaction stage. (3) Synthesized ATP and NADPH then participate Calvin cycle to generate sugar. Sugar catabolism (glycolysis and TCA cycle) provides the ATP and substrate (GA3P) required for carotenoid biosynthesis. (4) In addition, chloroplast Pi concentration changes regulate transcript abundances of carotenoid biosynthesis-limited genes PSY, PDS. Note: G6P, Glucose 6-phosphate; Xul-5p, xylulose 5-phosphate; PEP, phosphoenolpyruvic acid; TP, triose-phosphate; GA3P, glyceraldehyde 3-phosphate; IPP, isopentenyl diphosphate; DMAPP, dimethylallyl diphosphate; GGPP, geranylgeranyl diphosphate; PSY, phytoene synthase; PDS, phytoene desaturase; ABA, abscisic acids; ATP, adenosine triphosphate; ADP, adenosine diphosphate; TCA cycle, tricarboxylic acid cycle; BASS2, plastid-localized pyruvate transporter bile acid: sodium symporter family protein 2.

Orthophosphate (Pi) has multifaceted functions in plants (Chen et al., 2022). It serves as a substrate for ATP synthesis in photosynthesis and respiration (Millar et al., 2011; Junge and Nelson, 2015), and acts as a substrate for plastidic phosphate translocators, which participate in carbon assignment between starch and sucrose biosynthesis (Poirier et al., 2002; Rausch and Bucher, 2002; Linka and Weber, 2010), functions as an important component of NADPH, nucleic acids, sugar phosphates, DNA/RNA, and phospholipids in biological membranes (Xue et al., 2009), or modulates protein functions through phosphorylation by protein kinase (Romeis et al., 2001; Xue et al., 2009; Bayle et al., 2011; Chen et al., 2015). In addition, Pi functions as a component of phytate (Bohn et al., 2008; Wang et al., 2020) and serves as a structural cofactor in hormone perception (Tan et al., 2007; Sheard et al., 2010; Mosblech et al., 2011). Pi concentration variations in plastids affect ATP/ADP exchange velocity, sugar and starch metabolism processes (Carstensen et al., 2018). And these changes are expected to modulate pathways that are involved in carotenoid accumulation (details are described in **Figure 1** and related text).

Studies have shown that Pi homeostasis in plastids is controlled by several PHTs (López-Arredondo et al., 2014; Młodzińska and Zboińska, 2016), and manipulation of different plastid-derived PHTs has a distinct effect on carotenoid accumulation (Karlsson et al., 2015; Miyaji et al., 2015; Zhang et al., 2017; Lu et al., 2018). Therefore, this review summarizes plastid-located PHTs members, their functions in plastid Pi transport, their contributions to carotenoid accumulation, their underlying mechanism for this action, and a prospect to this field, anticipating facilitate the utilization of these PHTs for carotenoid accumulation through gene engineering.

## 2 Sub-cellular locations of phosphate transport proteins

**Figure 2:**
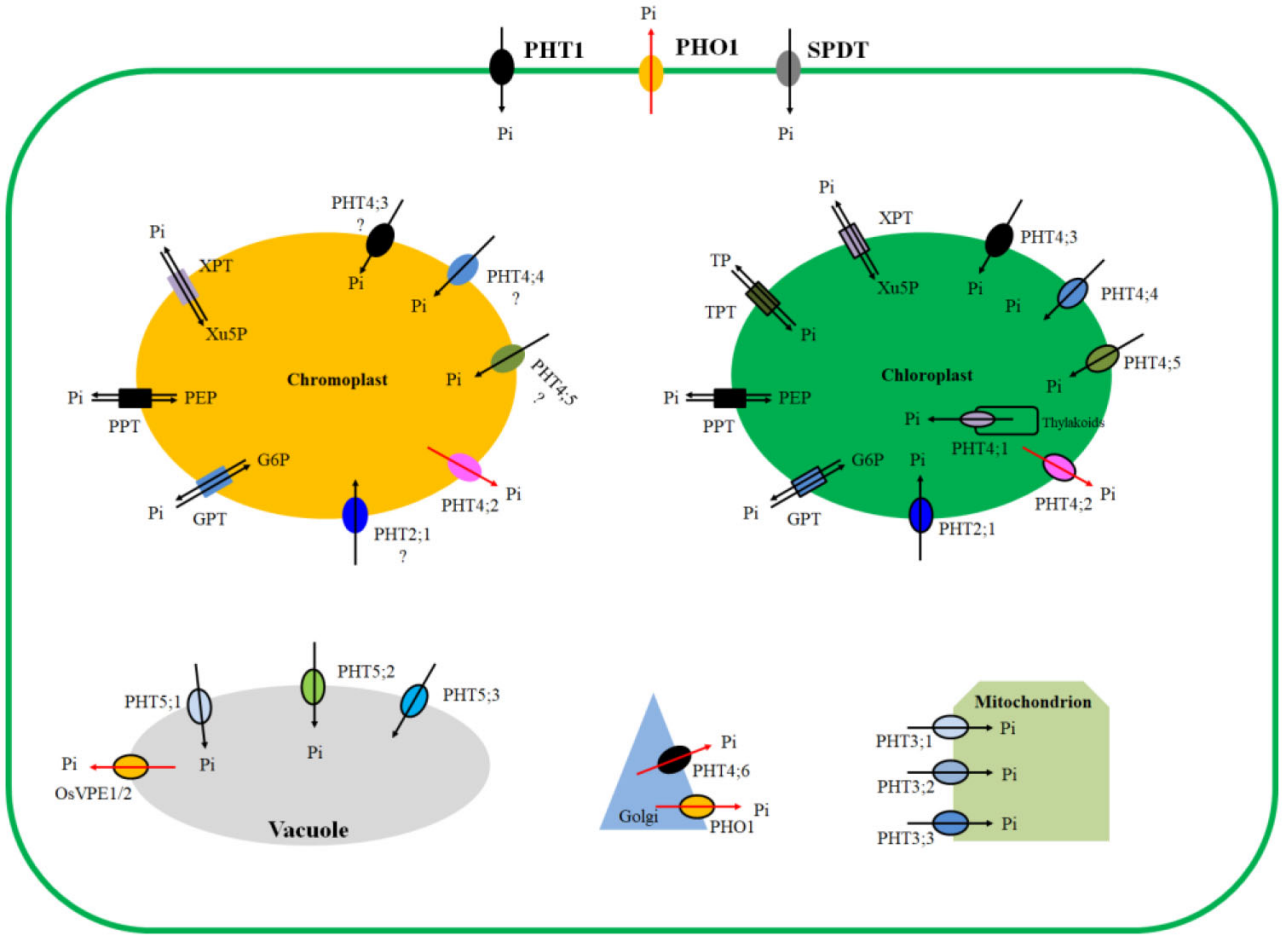
Subcellular locations of phosphate transport proteins. Plasma membrane located members: PHT1, PHO1, and SPDT. Golgi located members: PHT4;6 and PHO1. Mitochondrion located members: PHT3;1, PHT3;2, and PHT3;3. Vacuole located members: PHT5;1, PHT5;2, PHT5;3, and OsVPE1/2. Chloroplast located members: TPT, XPT, PPT, GPT, PHT2;1, PHT4;1, PHT4;2, PHT4;3, PHT4;4, PHT4;5. Chromoplast located members: XPT, PPT, GPT, PHT2;1, PHT4;2, PHT4;3, PHT4;4, PHT4;5. Chromoplast locations of PHT4;3, PHT4;4, PHT4;5 are proposed by speculation. Substrates transported are indicated by arrows. Note: PHT, phosphate transporter; PHO1, PHOSPHATE1; SPDT, sulfate transporter [SULTR]-like phosphorus distribution transporter; VPE, vacuolar phosphate efflux transporter; TPT, triose-phosphate/phosphate translocator; PPT, phosphoenolpyruvate/phosphate translocator; XPT, xylulose 5-phosphate/phosphate translocator; GPT, glucose 6-phosphate/phosphate translocator.

The exchange of phosphate or phosphorylated metabolites at cell and organelle levels is accomplished by specific phosphate transporters (PHTs), the plastidial phosphate translocator family (pPTs) of the inner envelope membrane, PHOSPHATE1 (PHO1), vacuolar phosphate efflux transporter (VPE), and Sulfate transporter [SULTR]-like phosphorus distribution transporter (SPDT) (Wang et al., 2017; Fabiańska et al., 2019; He et al., 2019; Victor Roch et al., 2019; Xu et al., 2019; Nguyen et al., 2021; Wang et al., 2021; Wang et al., 2021; Zhou et al., 2021; **Figure 2**). PHTs are grouped into five families: PHT1, PHT2, PHT3, PHT4, and PHT5 (Wang et al., 2017). Sub-cellular location results reveal that nine PHT1 are localized to the plasma membrane, three PHT3 are localized to the mitochondrion, and three PHT5 are localized to the vacuole. Except PHT4;6, which is localized to the Golgi, other five PHT4 and all PHT2 are localized to the plastid, especially to the carotenoid-enriched chromoplast and chloroplast (Finazzi et al., 2015; Młodzińska and Zboińska, 2016; Zhang et al., 2017; Wang et al., 2017). pPTs are grouped into four families: TPT (triose-phosphate/phosphate translocator), PPT (phosphoenolpyruvate/phosphate translocator), XPT (xylulose 5-phosphate/phosphate translocator), GPT (glucose 6-phosphate/phosphate translocator). TPTs are specifically localized to the chloroplast, whereas the other three pPTs are targeted to both the chloroplast and chromoplast (Fabiańska et al., 2019; Weise et al., 2019). PHO1s are targeted to both the plasma membrane and Golgi (Nguyen et al., 2021). VPEs are localized to the vacuole (Xu et al., 2019), and SPDTs are localized to the plasma membrane (Yamaji et al., 2017; Ding et al., 2020). It follows that amongst all phosphate transport proteins in plants, only plastid PHTs (PHT2, PHT4) and pPT are localized to the plastids (**Figure 2**). All pPT proteins catalyze a strict 1:1 exchange of sugar phosphates and inorganic phosphate, thereby guaranteeing the total phosphate balance of the plastid and the cytosol while allowing the transport of carbon and energy (Lee et al., 2017). PHTs affect various metabolic processes through their modulating of Pi homeostasis between cells and organelles (Mukherjee et al., 2015). Plastids are the site for carotenoid accumulation. Because manipulation of single plastid PHT enhances carotenoid accumulation (Zhang et al., 2017; Lu et al., 2018), whereas manipulation of either single or several pPTs does not affect carotenoid accumulation (Hilgers et al., 2018), this review focuses on the plastid PHTs (PHT4 & PHT2).

## 3 Roles of chromoplast-localized PHTs in carotenoid accumulation

### 3.1 Chromoplast-localized PHTs

Chromoplasts are specialized plastids found in some non-photosynthetic tissues of roots, flowers, fruits, and other carotenoid-accumulating tissues (Sun et al., 2018; Sadali et al., 2019). Amongst plastid-located PHTs in Arabidopsis and rice, PHT4;2 is the only member which is mainly expressed in non-photosynthetic tissues (root, fruit, flower, and other non-green organs), rather than expressed in photosynthetic tissues (leaf) (Daram et al., 1999; Guo et al., 2008a; Guo et al., 2008b; Irigoyen et al., 2011; Guo et al., 2012; Młodzińska and Zboińska, 2016; Versaw and Garcia, 2017; Zhang et al., 2017; Li et al., 2020). The expression position of PHT4;2 overlaps with tissues of chromoplast enrichment, suggesting a potential role of PHT4;2 in chromoplast Pi transport. Further subcellular localization experiments showed that ClPHT4;2 from *Citrullus lanatus* is targeted on the chromoplast (Zhang et al., 2017). To our knowledge, this protein is the only chromoplast-located PHT that is identified by direct experiment.

Western blot results showed that AtPHT4;2, an ortholog protein of ClPHT4;2, is detected in root plastid, rather than in leaf chloroplast (Irigoyen et al., 2011). The similar location pattern and high homology between them are tempting to speculate that AtPHT4;2 is also targeted on chromoplast, like ClPHT4;2. However, subcellular localization experiments using leaf protoplasts as expression hosts showed that expression signals of AtPHT4;2 and OsPHT4;2 were located on chloroplast (Guo et al., 2008a; Li et al., 2020). This phenomenon is partially attributed to the fact that chromoplast is almost absent in the leaves of many plants (Sadali et al., 2019; Llorente et al., 2020), resulting in signals of AtPHT4;2 and OsPHT4;2 are targeted to the chloroplast, an organelle with a similar structure to chromoplast. Hence, subcellular localization assays using protoplasts derived from heterotrophic tissues rather than autotrophic tissues, as in the assay for ClPHT4;2, are necessary to identify chromoplast-localized PHTs.

Of course, although they are mainly expressed in heterotrophic organs, transcripts of *OsPHT4;2* and *AtPHT4;2* are also detected in autotrophic tissues (Zhang et al., 2017; Li et al., 2020). The leaf chloroplast location of OsPHT4;2 (Guo et al., 2008a; Li et al., 2020) further supports the idea that PHT4;2 homologs are also chloroplast proteins, although with low abundances.

### 3.2 Functions in chromoplast phosphate transport

The observation that AtPHT4;2 (Guo et al., 2008a) and ClPHT4;2 (Zhang et al., 2017) mediate Pi uptake in yeast, demonstrates that they are both functional PHTs. Affinities for the substrate are in the low-affinity range, with Km of 0.5 mM and 0.44 mM, respectively (Guo et al., 2008a; Zhang et al., 2017). Knockout of AtPHT4;2 results in significantly reduced Pi export activity in root plastids (Irigoyen et al., 2011). Further pieces of evidence show that Pi export from chromoplast to the cytosol is a physiologically relevant role for PHT4;2 (Mukherjee et al., 2015). The above-mentioned results, in combination with the magnitude of ~60% reduction of Pi export (Irigoyen et al., 2011; Mukherjee et al., 2015), support the conclusion that PHT4;2 is a major Pi exporter in chromoplast.

### 3.3 Effects on carotenoid accumulation and underlying mechanism

Watermelon flesh carotenoid contents increase with increasing expression levels of ClPHT4;2, and knockdown of ClPHT4;2 reduces the fruit carotenoid accumulation (Zhang et al., 2017). A highly homologous plastid type PHT4;2 from *Citrus sinensis* (CsPHT4;2) is identified as closely correlated with high-lycopene accumulation induced by CPTA [2-(4-chlorophenylthio)-triethylamine hydrochloride]. Transient over-expression of CsPHT4;2 significantly enhances carotenoid accumulation in sweet orange juice vesi-cle-derived callus (Lu et al., 2018) (**Table 1**). In summary, it is proposed that overexpression of the chromoplast-located Pi exporter PHT4;2 enhances carotenoid accumulation, whereas knockout/knockdown of this gene reduces carotenoid accumulation in heterotrophic tissue.

**Table 1:** Effects of plastid phosphate transport genes transcript abundances changes on carotenoid and other secondary metabolites accumulation.

Gene name	Gene ID	Speciesorigin	Major location	Function	Affinityconstant	Effect on the carotenoidaccumulation	Effect on other secondary metabolites accumulation	References
***ClPHT4;2***	Cla017962	*Citrullus lanatus*	chromoplast in non-photosynthetic tissues	Pi export	440 μM	high carotenoid accumulation is closely correlated with a high transcriptional abundance of *ClPHT4;2*. Knockout down of *ClPHT4;2* leads to a reduction of carotenoid accumulation in non-photosynthetic tissues		Zhang et al., 2017
***CsPHT4;2***	Cs6g07670	*Citrus sinensis*	plastid in non-photosynthetic tissues	Pi export		Overexpression of *CsPHT4;2* enhances carotenoid accumulation in non-photosynthetic tissues		Lu et al., 2018
***AtPHT4;2***	At2g38060	Arabidopsis	Plastid in non-photosynthetic tissues	Pi export	510 μM			Guo et al., 2008a; Guo et al., 2008b; Irigoyen et al., 2011;
***AtPHT4;1***	At2g29650	Arabidopsis	chloroplast in photosynthetic tissues	Pi or ascorbate import	75 or 500 μM	Knockout of *AtPHT4;1* has no significant effect on the carotenoid accumulation in photosynthetic tissues	Knockout of *AtPHT4;1* suppresses salicylic acid accumulation	Guo et al., 2008a; Guo et al., 2008b; Pavon et al., 2008; Wang et al., 2014; Karlsson et al., 2015
***AtPHT4;4***	At4g00370	Arabidopsis	chloroplast in photosynthetic tissues	Pi import	720 μM	Knockout of *AtPHT4;4* has no significant effect on the carotenoid accumulation in photosynthetic tissues under low light but causes reduced carotenoid accumulation under high light.		Miyaji et al., 2015
***AtPHT2;1***	At3g26570	Arabidopsis	chloroplast in photosynthetic tissues	Pi import	400 μM			Versaw and Harrison, 2002
***TaPHT2;1***	AY293827	Wheat	chloroplast in photosynthetic tissues	Pi import	225 μM			Guo et al., 2012
***PvPHT2;1***	MT043283	*Pteris vittata*	chloroplast in photosynthetic tissues	Pi import				Feng et al., 2021
***OsPHT2;1***	LOC4329844	Rice	chloroplast in photosynthetic tissues	Pi import			Knockout of *OsPHT2;1* leads to reduced flavonoid accumulation	Liu et al., 2020
***AtXPT***	At5g17630	Arabidopsis	chloroplast in photosynthetic tissues and plastid in non-photosynthetic tissues	xylulose 5-phosphate/Pi exchange		Knockout of *AtXPT* does not affect the carotenoid accumulation in photosynthetic tissues		Hilgers et al., 2018
***AtPPT1/2***	At5g33320/At3g01550	Arabidopsis	chloroplast in photosynthetic tissues and plastid in non-photosynthetic tissues	phosphoenolpyruvate/Pi exchange		Knockout of *AtPPT* has no effect on carotenoid accumulation in photosynthetic tissues		Hilgers et al., 2018
***AtPPT1/2/AtXPT***	At5g17630/At5g33320/At3g01550	Arabidopsis	chloroplast in photosynthetic tissues and plastid in non-photosynthetic tissues	hexose phosphate/phosphate exchange		Double knockout of *AtXPT/PPT* does not affect the carotenoid accumulation in photosynthetic tissues		Hilgers et al., 2018

*PHT4;2* is the only currently known chromoplast gene that is responsible for Pi efflux. Knockdown of *PHT4;2* results in an accumulation of chromoplast Pi concentration, whereas upregulation of *PHT4;2* leads to a reduction of chromoplast Pi concentration (**Figure 1A**). The variation of chromoplast Pi upon *PHT4;2* transcript abundances changes is proposed to regulate carotenoid accumulation through the following pathways.

Firstly, various biosynthetic processes occurring in non-photosynthetic plastids require hydrolysis of ATP to provide energy and timely export of Pi from chromoplast is crucial for the ATP hydrolysis reaction. As a major contributor to this export activity (Irigoyen et al., 2011; Versaw and Garcia, 2017), overexpression of PHT4;2 leads to accelerated Pi efflux from chromoplast, facilitating ATP hydrolysis to provide energy, and then accelerates sugar catabolism, facilitating the synthesis of glyceraldehyde 3-phosphate (GA3P), a substrate required for carotenoid biosynthesis (**Figure 1A**).

Secondly, this accelerated Pi movement promotes ATP synthesis by accelerating the sugar catabolism process (glycolysis and tricarboxylic acid cycle) (**Figure 1A**). ATP synthase and adenine nucleotide translocator represent two of the most highly abundant proteins in chromoplast proteomes of various crops (Wang et al., 2013), and glycolytic and oxidative energy metabolism is enhanced during chromoplast differentiation (Llorente et al., 2020), supporting that enhanced energy production and transport facilitates chromoplast development. The various carotenoid-lipoprotein sequestering substructures of chromoplast play crucial roles in massive accumulation in chromoplasts through sequestering the newly synthesized carotenoids into pigmentlipoprotein substructures within chromoplasts for stable storage, and through stimulating continuous biosynthesis by removing the newly synthesized carotenoids from plastid envelope membranes to avoid overloading of endproducts at the site of carotenoid biosynthesis (Vishnevetsky et al., 1999; Merzlyak and Solovchenko, 2002; Li L. et al., 2016). The enhanced energy supply favored by PHT4;2 overexpression is thus beneficial to chromoplast development (Li and Yuan, 2013; Zhang et al., 2017) and then facilitates carotenoid storage (**Figure 1A**).

Thirdly, a reduction of Pi concentration in chromoplast achieved by the enhancement of ClPHT4;2 causes the promotion of key carotenoid biosynthetic genes such as *ClPSY* and *ClPDS*, finally enhancing carotenoid accumulation in plants (Zhang et al., 2017). The boost of Pi concentration in chromoplast caused by ClPHT4;2 knockdown leads to the inhibition of carotenoid biosynthetic genes such as *ClPSY* (phytoene synthase) and *ClPDS* (phytoene desaturase), finally suppressing the carotenoid biosynthetic pathway (Zhang et al., 2017; **Figure 1A**). This case is in accordance with the observation that external phosphate starvation causes Pi limitation in the plastid and upregulates these key carotenoid biosynthetic genes (Fantini et al., 2013; Walter et al., 2015; Sathasivam et al., 2020), whereas high Pi treatment causes the accumulation of Pi mainly in the plastid (Ryan et al., 2019) and represses the transcript abundances of rate-limiting carotenoid biosynthetic genes such as *PSY3* (Breuillin et al., 2010; Lu et al., 2021) (**Figure 1A**).

## 4 Roles of chloroplast-localized PHTs in carotenoid accumulation

### 4.1 Chloroplast-localized PHTs

Except PHT4;2, the other four plastid-located PHT4s (PHT4;1, PHT4;3-PHT4;5) share a similar expression pattern. They are mainly detected in autotrophic tissues (leaf), rather than in heterotrophic tissues (root, fruit, flower, et ac), suggesting potentially critical roles of these PHTs in leaf plastid Pi transport (Guo et al., 2008a; Li et al., 2020). Further subcellular localization experiments show that AtPHT4;1, AtPHT4;4, and AtPHT4;5 are targeted to the leaf chloroplast (Pavon et al., 2008; Ferro et al., 2010; Miyaji et al., 2015; Yin et al., 2015). In detail, AtPHT4;1 is localized to chloroplast thylakoid membranes, whereas AtPHT4;4 and AtPHT4;5 are localized to the chloroplast envelope (Roth et al., 2004; Miyaji et al., 2015; Yin et al., 2015). AtPHT4;3 is localized to shoot plastid, with the situation of its precise plastid category remaining unclear (Guo et al., 2008a). A highly homologous protein OsPHT4;3 is localized to leaf chloroplast (Li et al., 2020), supporting the speculation that AtPHT4;3 is also targeted to leaf chloroplast, although direct experimental evidence is still required. Hence, except PHT4;2, the other four PHT4s are proposed to be localized to leaf chloroplast, supporting their functions in chloroplast Pi transport.

Like above mentioned four PHT4s, PHT2 is mainly detected in autotrophic leaves (Versaw and Harrison, 2002; Liu et al., 2011; Guo et al., 2012; Zhang et al., 2016; Liu et al., 2020; Feng et al., 2021). Arabidopsis AtPHT2;1, wheat TaPHT2;1, *Pteris vittata* PvPHT2;1, rice OsPHT2;1, *Medicago truncatula* MtPHT2;1 and *P. simonii* PtrPHT2;1 and PtrPHT2;2 are targeted to leaf chloroplast (Versaw and Harrison, 2002; Zhao et al., 2003; Liu et al., 2011; Guo et al., 2012; Zhang et al., 2016; Liu et al., 2020; Feng et al., 2021).

It is worthy to note that besides autotrophic tissues, transcripts of *PHT4;1*, *PHT4;3*, *PHT4;4*, *PHT4;5*, and *PHT2;1* are also found in heterotrophic tissues (although with low transcript abundances), supporting the speculation that these PHTs potentially function in heterotrophic tissues plastid (such as chromoplast, amyloplasts) Pi transport, and they might coordinate with PHT4;2 to modulate Pi homeostasis in chromoplast (Guo et al., 2008b; Fabiańska et al., 2019).

### 4.2 Functions in chloroplast phosphate transport

PHT4;1 and PHT4;2-4;5 (except OsPHT4;5) from Arabidopsis and rice rescue the growth of yeast defective in Pi uptake upon restricted Pi supply, demonstrating that they are capable of mediating Pi transport (Guo et al., 2008a; Li et al., 2020). Knockout of AtPHT4;1 in Arabidopsis leads to a reduced ATP synthase activity in the chloroplast, which is attributed to a decreased supply of Pi in the stroma (Karlsson et al., 2015). Both AtPHT4;1 and AtPHT4;4 are assumed to directly modulate Pi concentration in the chloroplast (Finazzi et al., 2015; Karlsson et al., 2015). However, direct measurement of Pi concentration changes in chloroplast caused by the absence of these PHTs is still lacking.

Except for being a phosphate transport protein, AtPHT4;4 functions as an ascorbate transporter, and is responsible for transporting the reduced form of ascorbate synthesized by mitochondria into chloroplast to neutralize high light damage (Miyaji et al., 2015; Nam et al., 2021). AtPHT4;4 possesses the ability to transport ascorbate, whereas AtPHT4;1 and AtPHT4;3 cannot transport this reagent (Karlsson et al., 2015; Miyaji et al., 2015).

As for PHT2, Arabidopsis AtPHT2;1, wheat TaPHT2;1, *Pteris vittata* PvPHT2;1, and rice OsPHT2;1 have been functionally characterized in the chloroplast envelope as low-affinity proton/Pi symporters (Versaw and Harrison, 2002; Guo et al., 2012; Liu et al., 2020; Feng et al., 2021). Knockdown expression of TaPHT2;1 leads to a significantly reduced Pi concentration in the chloroplast, indicating that TaPHT2;1 is crucial for the translocation of Pi from the cytosol to the chloroplast (Guo et al., 2012). Overexpression of PvPHT2;1 in Arabidopsis causes a 37−59% increase in chloroplasts’ Pi content (Feng et al., 2021). It is worthy to note that under normal Pi supply, knockout or overexpression of single chloroplast PHT does not affect the leaf Pi content (Karlsson et al., 2015; Liu et al., 2020). All these results demonstrate that chloroplast PHTs are involved in the chloroplast Pi influx, and functional redundancy exists amongst these PHTs.

### 4.3 Functions in carotenoid accumulation and underlying mechanism

Knockout of either AtPHT4;1 or AtPHT4;4 has no significant impact on the contents of several major carotenoids, namely β-carotene, violaxanthin, and lutein in Arabidopsis leaves (Karlsson et al., 2015; Miyaji et al., 2015) (**Table 1**). Although these several major carotenoids are reduced by AtPHT4;4 knockout under high light, this phenomenon is attributed to its ascorbate transport activity (xanthophyll cycle is suppressed), rather than to its Pi transport activity (Miyaji et al., 2015). In chloroplasts, the concentration and composition of carotenoids are relatively constant for the functions of light harvesting and photoprotection (Hermanns et al., 2020). Simultaneously, a precise balance of chlorophylls and carotenoids is required for photosynthesis (Domonkos et al., 2013; Esteban et al., 2015; Hashimoto et al., 2016; Andersen et al., 2020). Knockout of either AtPHT4;3 or AtPHT4;5 has no significant impact on the chlorophyll contents (Nam et al., 2021), suggesting that carotenoid accumulation is also unaffected by these two genes. As for PHT2, the fact that no different carotenoid metabolism is detected between wild type and pht2;1 knockout rice plants (Liu et al., 2020) demonstrates that knockout of PHT2;1 also does not affect the carotenoid accumulation. Taken together, the manipulation of single chloroplast-located PHT did not significantly affect the carotenoid content in leaves (Karlsson et al., 2015; Miyaji et al., 2015). The underlying mechanism of this phenomenon may be the result of the following reasons.

Functional redundancy amongst chloroplast PHTs leads to the consequence that manipulation of a single chloroplast PHT has no substantial impact on the chloroplast Pi concentration. This unchanged chloroplast Pi concentration upon single PHT absence is thus unable to regulate Pi-involved carotenoid accumulation processes and modulate carotenoid accumulation (**Figure 1B**). Pieces of evidence are listed as follows. i) chloroplast phosphate homeostasis plays a crucial role in photosynthesis by maintaining ATP synthesis and driving the Calvin cycle (Mimura et al., 1990), and affects the energy requirement of various metabolic processes (including carotenoid, sugar, and starch metabolism) occurring in plastids through modulating ATP/ADP exchange rate (**Figure 1B**). ii) Pi levels in chloroplasts are controlled by at least five chloroplast Pi transporters (López-Arredondo et al., 2014; Młodzińska and Zboińska, 2016). Knockout of single chloroplast PHT does not affect the leaf Pi content under normal conditions (Karlsson et al., 2015; Liu et al., 2020). iii) ATP synthase activity is very sensitive to changes in the chloroplast Pi concentration and even minor reductions in stromal Pi concentration have a major influence on ATP synthase activity (Carstensen et al., 2018). Pi limitation in chloroplast initially causes suppression of ATP synthase activity, and subsequently, inhibition of the process for proton efflux from the thylakoid lumen to the chloroplast stroma, finally leading to lumen acidification. Pi-resupply reverses this phenomenon and abolishes lumen acidification (Carstensen et al., 2018). Knockout of PHT4;1 leads to lumen acidification in the first 2-3 min, and this lumen acidification disappears when the time extends to 10 min (Karlsson et al., 2015). In addition, no lumen acidification is detected in the PHT4;4 knockout plants (Miyaji et al., 2015). All these results demonstrate that other chloroplast PHTs compensate for the Pi limitation caused by PHT4;1/PHT4;4 absence, allowing the concentration in chloroplast to restore to the wild-type level. The compensation of chloroplast Pi concentration is proposed to be achieved by either transcript upregulation or protein modification of other plastid PHTs (Bayle et al., 2011; Chen et al., 2015; Yang et al., 2020; Wang et al., 2020). iv) Considering that PHT4;1/PHT4;4 absence has no substantial effect on the chloroplast Pi concentration, the mutations are thus unable to modulate the Pi-involved pathways that involve carotenoid accumulation (**Figure 1B**). The co-expression pattern of these chloroplast PHTs is similar to the case of plasma membrane ammonium transporters, which use a functional redundancy among three major ammonium transporters to coordinate ammonium transport (Yuan et al., 2007; Li C. et al., 2016; Hao et al., 2020a; Hao et al., 2020b; Konishi and Ma, 2021). Taken together, it is proposed that each chloroplast PHT contributes to net Pi flux across the chloroplasts (López-Arredondo et al., 2014), and functional redundancy occurs amongst the five ones, consequently leading to the inability of manipulating a single chloroplast PHT gene to affect carotenoid accumulation. In addition, the short-time (< 2min) Pi concentration changes upon single chloroplast PHT mutation seemingly do not affect the un-well known network that maintains the relatively constant concentration and composition of carotenoids, which are essential for photosynthesis (Domonkos et al., 2013; Esteban et al., 2015; Hashimoto et al., 2016; Andersen et al., 2020; Hermanns et al., 2020).

## 5 Roles of plastid-located PHTs in other secondary metabolites accumulation

Chloroplast is the major site for salicylic acid biosynthesis and is capable of flavonoid biosynthesis (Agati et al., 2007; Wang et al., 2011). The absence of chloroplast AtPHT4;1 leads to a suppression of salicylic acid accumulation in a small gain-of-function mutant that displays extreme dwarfism, constitutive defense, and spontaneous cell death phenotypes (Wang et al., 2011; Wang et al., 2014), whereas knockout of chloroplast OsPHT2;1 results in a reduction of flavonoid accumulation (Liu et al., 2020). These findings provide new ideas for enhancing these secondary metabolite accumulations.

## 6 Conclusions and prospects

Chloroplast in photosynthetic tissues and chromoplast in non-photosynthetic tissues are the two major organelles of carotenoid accumulation (Ruiz-Sola and Rodrı´guez-Concepcio´n, 2012; Li L. et al., 2016; Sun et al., 2018; Hermanns et al., 2020). Recently, manipulation of a chromoplast PHT, rather than those conventional genes which directly involve carotenoid biosynthesis or storage, enhances carotenoid accumulation in plants (Zhang et al., 2017; Lu et al., 2018). This advance provides a new idea for comprehensive carotenoid enrichment. Surprisingly, different plastid-derived PHTs show distinct (“have” or “no”) effects on carotenoid accumulation. By concentrating on chromoplast and chloroplast PHT members, their contributions to plastid Pi transport, their influence on carotenoid accumulation, and the underlying mechanism for modulating carotenoid enrichment, this review summarizes the roles of plastid PHTs in carotenoid accumulation and makes a prospect, with anticipation to facilitate the utilization of these transporters for carotenoid enrichment. Conclusions are drawn as follows.

(i) Amongst all phosphate transport proteins in plants, only plastid PHTs (PHT2 & PHT4) and pPT are localized to the plastid (**Figure 2**). More interestingly, they are all located on the plastid of carotenoid accumulation, that is, chromoplast in non-photosynthetic tissues and chloroplast in photosynthetic tissues, suggesting that they may play roles in regulating carotenoid accumulation. Given that knockout of either single or several pPT does not affect the carotenoid accumulation, whereas manipulation of single plastid PHTs modulates carotenoid accumulation (**Table 1**), this review focuses on the plastid PHTs.

(ii) One chromoplast PHT (PHT4;2) and at least five chloroplast PHTs (PHT2;1, PHT4;1, PHT4;3, PHT4;4, PHT4;5) are identified in plants. Manipulation of single chromoplast PHT modulates the carotenoid accumulation in non-photosynthetic tissues, whereas manipulation of single chloroplast PHT has no significant impact on the carotenoid accumulation in photosynthetic tissues. The chromoplast PHT is thus proposed to function in enhancing carotenoid accumulation in plants whose non-photosynthetic tissues are harvested. In addition, the simultaneous promotion of several kinds of carotenoids conferred by chromoplast PHT overexpression is beneficial for diverse carotenoid demands of humans intaken from the diet.

(iii) The underlying mechanism for the observation that manipulation of single chromoplast PHT modulates the carotenoid accumulation may be attributed to the following reasons. PHT4;2 is the only chromoplast Pi exporter, and manipulating this single chromoplast PHT significantly regulates chromoplast Pi concentration. This variation subsequently modulates the carotenoid accumulation by affecting the supply of a substrate (glyceraldehyde 3-phosphate) for carotenoid biosynthesis, by modulating the transcript abundances of carotenoid biosynthesis limited enzyme genes, and by regulating chromoplast biogenesis (facilitating carotenoid storage) (**Figure 1A**).

(iv) The underlying mechanism for the observation that manipulation of single chloroplast PHT has no significant impact on the carotenoid accumulation may be the results of the following reasons. At least five Pi influx PHTs are identified in the chloroplast, and manipulating one of the five does not substantially modulate the chloroplast Pi concentration in a long term due to their functional redundancy. This unchanged chloroplast Pi concentration upon one chloroplast PHT absence, therefore, is unable to modulate Pi-involved carotenoid accumulation processes and finally does affect carotenoid accumulation in photosynthetic tissues (**Figure 1B**).

Despite these advances, further investigation is needed for the utilization of plastid PHTs for carotenoid enhancement through gene engineering. We believe that several items listed below should be taken with caution.

(i) Precise Location of PHTs in Plastid

Chloroplast in photosynthetic tissues and chromoplast in non-photosynthetic tissues are the two major organelles of carotenoid accumulation. And manipulation of different organelles-derived PHTs has a different effect on carotenoid accumulation, indicating that elucidation of the precise location of these transporters, whether chloroplast or chromoplast, is important and necessary. Since many plant leaves do not have chromoplasts (Sadali et al., 2019; Llorente et al., 2020), inappropriate interpretation of the subcellular localization of these PHTs occurs if just using leaf protoplasts as expression hosts to investigate the subcellular localization of those chromoplast located PHTs. Hence, not only protoplasts from photosynthetic tissues (enrich in the chloroplast) but also protoplasts from non-photosynthetic tissues (enrich in chromoplast) should be used as expression receptors when carrying subcellular localization assays of plastid-located PHTs, ensuring the precise location of these PHTs. Given that location pattern is closely correlated with their physiological functions, the precise location of these PHTs should be taken with caution.

(ii) The Phosphate Transport Direction Mediated by Plastid PHTs

Although the ability to transport Pi has been identified in the yeast system, the Pi transport direction (influx or efflux) mediated by these plastid PHTs still needs further investigation. By comparisons of plastid Pi content changes before and after their absence, the direction of Pi transport across the plastid has been clarified for PHT4;2 and PHT2;1. Despite high homology, the direction of Pi transport undertaken by them is opposite (efflux vs influx), demonstrating that using a heterologous system such as yeast to study the physiological role of these plastid PHTs *in planta* is unsuitable. Therefore, it is necessary to clarify the direction of Pi transport in plastid by direct measurement of Pi content changes upon overexpression and/or knockout of these PHTs *in planta*, facilitating clarification of their physiological roles in carotenoid accumulation. Several amino acids and even single amino acid mutation of potassium channels/ammonium transporters lead to the reverse of potassium transport direction and/or inability to transport ammonium (Porée et al., 2005; Li et al., 2008; Hao et al., 2016; Huang et al., 2021), providing clues for understanding the case that highly homologous plastid PHTs have opposite substrate transport direction.

(iii) Win-Win on Both Sides of Yield and Carotenoid Accumulation, as Well as Potential Roles of Plastid PHTs in Leaf Carotenoid Accumulation

Although manipulation of single chromoplast PHT enhances carotenoid accumulation in non-photosynthetic tissues, whether it leads to a yield penalty has not been reported. Win-win on both sides of yield and carotenoid accumulation through gene engineering of plastid PHTs is a prerequisite for its use in the field.

Overexpression of single chromoplast PHT enhances carotenoid accumulation in non-photosynthetic tissues, whereas manipulation of single chloroplast PHT has no significant effect on the carotenoid accumulation in photosynthetic tissues. Given that carotenoid accumulation in photosynthetic tissues is an important index for the quality of leaf vegetables, analysis of double and even several chloroplast PHTs knockout/overexpression lines is necessary for clarifying their roles in leaf carotenoid accumulation. It is worthy to notice that the information regarding the carotenoid accumulation in photosynthetic tissues is lacking in the course of chromoplast PHT knockout/overexpression lines analysis, although carotenoid accumulation in non-photosynthetic tissues is measured. Similarly, the information regarding the carotenoid accumulation in non-photosynthetic tissues is lacking in the course of chloroplast PHT knockout/overexpression lines analysis, although carotenoid accumulation in photosynthetic tissues is investigated. Hence, whether the manipulation of single plastid PHT affects the total carotenoid accumulation in whole plants, both in non-photosynthetic tissues and photosynthetic tissues, needs further investigation. Given that photosynthetic parts of some plants are edible, whereas non-photosynthetic parts of other plants are edible by humans, answering the above question is crucial for the utilization of these transporters for tissue-specific carotenoid accumulation enhancement.

(iv) Roles in Accumulation of Other Secondary Metabolites and Underlying Mechanism

Besides carotenoids, manipulation of plastid PHTs can modulate the accumulation of other secondary metabolites, such as flavonoids and salicylic acid, providing a new strategy for enhancing these and even much more kinds of secondary metabolites (**Table 1**). However, the underlying mechanism needs further investigation. Additionally, the underlying mechanism for Pi-involved chromoplast biogenesis awaits research.

## Author contributions

D-LH and J-YZ designed the conceptualization and prepared the draft manuscript. Y-NH, H-RW, X-HL, H-LG, and J-XL did the formal analysis and revised the manuscript. All authors contributed to the article and approved the submitted version.
